# RELATIONSHIP BETWEEN THE USE OF KANGAROO POSITION ON PRETERM BABIES AND MOTHER-CHILD INTERACTION UPON DISCHARGE

**DOI:** 10.1590/1984-0462/;2017;35;2;00006

**Published:** 2017

**Authors:** Cynthia Ribeiro do Nascimento Nunes, Luís Gustavo Campos, Aline Moreira Lucena, Janser Moura Pereira, Patrícia Rodrigues da Costa, Flávia Aparecida Felipe de Lima, Vivian Mara Gonçalves de Oliveira Azevedo

**Affiliations:** a Universidade Federal de Minas Gerais (UFMG), Belo Horizonte, MG, Brasil.; b Hospital Sofia Feldman, Belo Horizonte, MG, Brasil.; c Universidade Federal de Uberlândia, Uberlândia, MG, Brasil.

**Keywords:** mother-child relations, premature infant, newborn low weight, kangaroo care, language, growth and development

## Abstract

**Objective:**

To analyze the influence of the Kangaroo Position duration in the initial interactions between mothers and preterm infants.

**Methods:**

This is an exploratory prospective observational study that analyzed the mother-infant interaction during breastfeeding, before hospital discharge. All eligible newborns, with a gestational age of 28-32 weeks and a birth weight of 1,000-1,800 g from June 11 to September 31, 2014 were included. The films of the interaction were evaluated by the “Mother-Baby Interaction Protocol 0-6 months” tool. The duration of the Kangaroo Position during all the hospitalization period was correlated with the interaction between mother and preterm infant.

**Results:**

The longer the dyad spent time in the Kangaroo Position, the more the newborns made physical contact attempts with their mothers during breastfeeding (r=0.37; *p*=0.03); and the longer the time in the Kangaroo Position, the less the mothers talked to their children (r=-0.47; *p*=0.006).

**Conclusions:**

Longer periods in the Kangaroo Position stimulates the initial exchanges of contact between preterm infant with his mother, which suggests a higher alert status of the newborn and a better availability for interactions with the mother during breastfeeding.

## RESUMO

**Objetivo:**

Analisar a influência da duração da Posição Canguru nas interações iniciais da díade mãe-filho pré-termo.

**Métodos:**

Trata-se de um estudo observacional, prospectivo exploratório, que analisou, por meio de filmagens, a interação mãe-filho, durante a amamentação, previamente à alta hospitalar, utilizando o “Protocolo de Interação Mãe-Bebê 0 a 6 meses”. Foi correlacionado o tempo da Posição Canguru durante toda a internação hospitalar e a interação mãe-filho pré-termo. Todos os recém-nascidos elegíveis (idade gestacional entre 28 e 32 semanas e peso ao nascimento entre 1000 e 1800 g) foram incluídos no período de 11 de junho a 31 de setembro de 2014.

**Resultados:**

Quanto maior o tempo em Posição Canguru, mais os recém-nascidos realizaram tentativas de contato físico com as mães durante a amamentação (*r*=0,37; *p*=0,03); e quanto maior o tempo na Posição Canguru, menos as mães conversaram com os filhos (*r*=-0,47; *p*=0,006).

**Conclusões:**

Os dados apontam que o maior tempo de Posição Canguru favorece as trocas iniciais de contato entre o filho pré-termo e a mãe, o que sugere maior estado de alerta e melhor disponibilidade do recém-nascido para interações com a mãe durante a amamentação.

## INTRODUCTION

Preterm newborns are considered a risk group for neuropsychomotor development and may present disadvantages in establishing satisfactory interactions,^1^ as well as delays in language acquisition and development.^2^ ^,^ ^3^ The newborn, who needs to remain in intensive care, can generate feelings of guilt and sadness in the mother, hindering the emergence of maternal sensitivity.^4^

In order to minimize the negative effects of neonatal hospitalization, the Kangaroo Method, introduced in Brazilian health units since the 1990s, has been incorporated into health policies and inserted in the context of the humanization of neonatal care, which includes the Kangaroo Position. It consists of keeping the low-weight newborn skin-to-skin, upright, close to the chest of the parents or other family members. It should be performed in an orientated, safe, and monitored manner by a properly trained health team.^5^

Among the benefits of the Kangaroo Method are: lower mortality risk,^6^ physiological stability, shorter hospital stay, stronger mother-child dyad interaction,^7^ improved maternal mood states,^8^ and increased maintenance of breastfeeding.^9^

The first exchanges between mother and child as demonstrated by emotional behavior - vocalizations, looking at the mother, reactivity and readiness to interact^10^ - are considered to be the source of the emergence and development of social and emotional skills, attachment construction, and language acquisition.^11^ The Kangaroo Position seems to influence the development of mother--child interaction (since the pre-verbal period) and maternal sensitivity (during hospital stay). However, the literature does not yet clearly describe how these initial interactions occur in preterm newborns who stayed in the Kangaroo Position and, especially, whether the duration of this position influences the mother-child dyad interaction.

In this context, this study aimed at analyzing the influence of the duration of the Kangaroo Position, on the initial interactions of the preterm infant-mother dyad.

## METHOD

This is an observational, prospective, exploratory study that adopted a non-probabilistic sample. Preterm newborns with gestational age between 28 and 32 weeks, and birth weight between 1,000 and 1,800 g, born at Hospital Sofia Feldman between June 11 and September 31, 2014, who remained in a neonatal unit in this institution and progressed to exclusive or mixed breastfeeding were included. The exclusion criteria were newborns with severe neurological impairment, such as intraventricular hemorrhage (grades III and IV), and leukomalacia; Apgar score at 5 minutes lower than seven; mothers who gave up due to discomfort with filming; mother-infant dyads who did not record and/or lost the record of the frequency and duration of the Kangaroo Position during hospital admission; and newborns who did not progress to breastfeeding.

**Figure 1:**
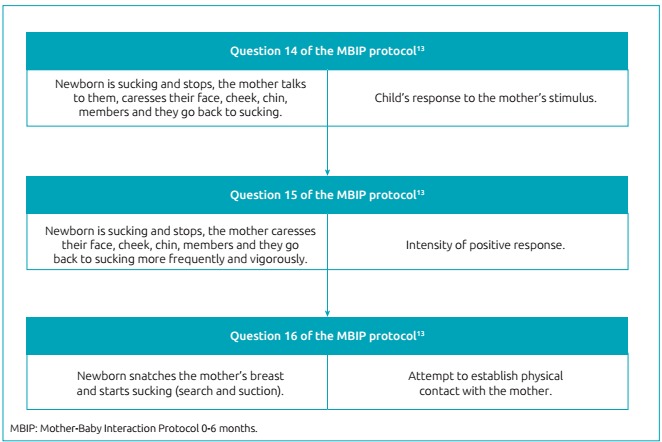
Criteria adapted to the situation of breastfeeding in the evaluation by the Mother-Baby Interaction Protocol 0-6 months (MBIP).^13^

This study was carried out at Hospital Sofia Feldman, a philanthropic institution that currently performs, on average, 900 births per month.^12^ Data collection was carried out in five phases:

- PHASE 1: A daily active search was performed in order to find newborns that met the inclusion criteria of this study. Soon after reading the medical record, clarification of possible doubts, acceptance of participation in the research and the signing of the informed consent, the mother-child dyad was included in the study, and monitored until hospital discharge. In this approach, a record form of Kangaroo Position, with information about the day, frequency, and duration of the position, was affixed to the incubator and both the team and the mother and the family were instructed on its completion. It is noteworthy that all newborns included in this study went through the three steps described by the Kangaroo Method.
- PHASE 2: Upon discharge from the Speech-Language Pathologist, when the newborn was fed exclusively orally, the research team approached the mother and completed the questionnaire elaborated for this research, with personal and socioeconomic data (address and telephone, maternal age, social conditions - income, maternity leave, family life), as well as data on gestational history, previous knowledge about the Kangaroo Method, experience with breastfeeding and relationship with the child in the delivery room.
- PHASE 3: Between the discharge from the Speech-Language Pathologist and hospital discharge, newborns who progressed to exclusive or mixed breastfeeding were filmed for three minutes of mother-infant interaction during breastfeeding. Filming was performed in the hospital bed itself and the camera was placed on a support (tripod), adjusted about 90 cm in height, so that it focused both the son’s and the mother’s responses and obtained the best lateral angle.
- PHASE 4: Two examiners with experience and performance in Neonatology were previously trained to perform analyses of the initial interaction videos through the Mother-Baby Interaction Protocol 0-6 months (MBIP 0-6).^13^ This instrument^13^ consists of 20 items, 11 of which refer to the behavior of the mother, 8 to the behavior of the newborn, and one to the behavior of the dyad. The mother’s items are categorized into: sensitivity, intrusiveness, and maternal involvement. Each of the items in the protocol has five score options from one to five, in ascending order, from the least satisfactory to the most satisfactory, with a detailed description of the corresponding behavior. In a consensus among the video evaluators and with support from the literature, in order to adapt the feeding situation, sucking was considered as a form of physical contact between the son and the mother. Figure 1 illustrates the criteria adapted to the breastfeeding situation. The evaluators were blinded as to the status of the frequency and duration of the Kangaroo Position of the mother-child dyad, in order to soften the judgment. A test phase was performed and the Kappa coefficient was applied, which obtained a value of 0.72, which suggests a good or substantial agreement between the evaluators.
- PHASE 5: The videos were filmed using a Samsung camcorder, Model S630. For editing, Windows Movie Maker software version 2014 was used, so that all videos had about the same duration. Audacity software version 2012 was used to soften the high volumes of environmental noise. The initial 20 seconds, during which the researcher was still present guiding the mother about the procedure, were excluded. This was to ease the bias due to the fact that, initially, the camera could inhibit the action of the mothers until they became accustomed to its presence.

In this study, univariate and bivariate analyses were used. The purpose of the univariate analysis was to characterize the sample using descriptive statistics. The bivariate analysis evaluated the linear correlation from the Pearson or Spearman linear correlation coefficients. The data collected were tabulated in a 2007 Microsoft Office Excel spreadsheet, and to analyze the association/correlation between two variables, the Pearson (r) linear correlation coefficient was used, which gives the degree and direction of the linear association between two variables. If r=0, the variables are not linearly correlated; if r>0, there is a positive linear relationship; if r<0, there is a negative linear relationship. It should be noted that all statistical analyses were performed in freeware *R*.^14^

This study was approved by the Research Ethics Committee of the Hospital Sofia Feldman (HSF), under protocol no. 668.309.

## RESULTS

**Figure 2:**
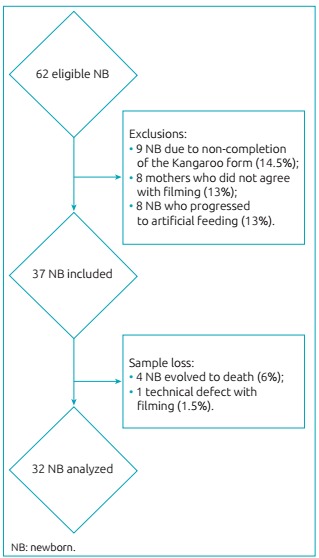
Flowchart with the distribution of the eligible sample, the losses, and the sample analyzed.

We analyzed 32 videos of initial mother-child interaction. Figure 2 shows the flowchart with the distribution of the eligible sample, the exclusion criteria, the losses, and the final sample analyzed. Twenty-nine mothers (of whom 3 are mothers of twins) and 32 newborns participated in this study. Among these mothers, 18 women (62.0%) were primigravidae and the others (38.0%) varied from the second to the eighth gestation. Seventeen (53.12%) newborns were delivered with normal delivery, and 15 (47.87%) were delivered with cesarean sections. Only 2 mothers (6.8%) reported knowing the Kangaroo Method before the current hospitalization, none of them breastfed the child in the delivery room, and only one (3.4%) reported having skin-to-skin contact with the child in the delivery room. Table 1 shows characteristics related to the maternal context.

**Table 1:**
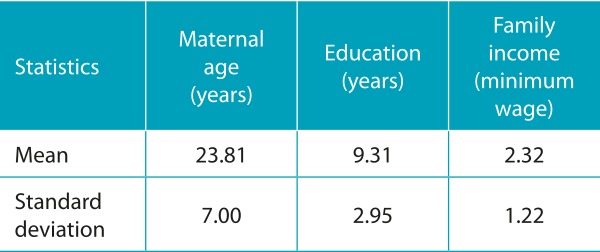
Characterization of the maternal context.

In relation to newborns, 17 (53.0%) were males. Table 2 presents the characteristics of newborns and the performance of the Kangaroo Position.

**Table 2:**
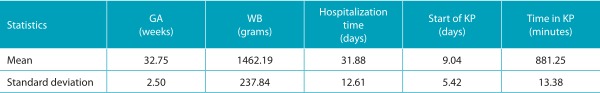
Characterization of the newborns and performance of the Kangaroo Position.

GA: gestational age; WB: weight at birth; KP: Kangaroo Position.

When excluding the dyads that did not perform the Kangaroo Position, the minimum frequency of this positioning was 1 time and the maximum was 21 times. Regarding the duration of the Kangaroo Position, the minimum total time was 60 minutes and the maximum was 4,290 minutes (about 70 hours). The hospital stay ranged from 14 to 70 days. Therefore, the longer the hospital stay, the more hours the mothers performed the Kangaroo Position (*r*=0.36; *p*=0.01).

Table 3 shows the results of the Pearson linear correlation between the duration of the Kangaroo Position and the scores of MBIP items.

**Table 3:**
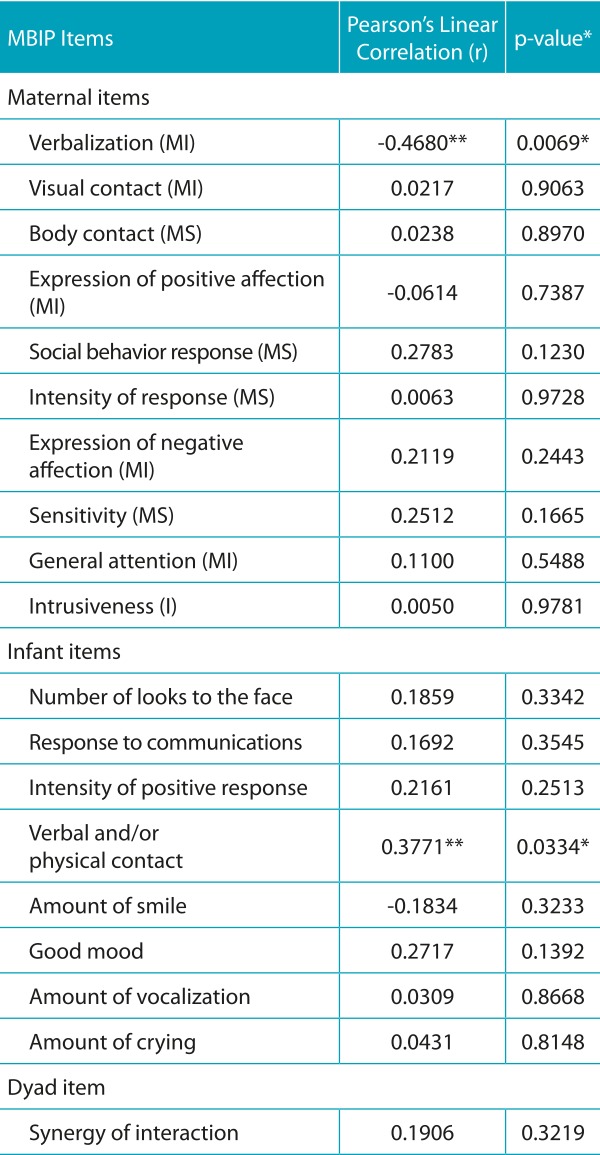
Correlation between the duration of the Kangaroo Position and the scores of the Mother-Baby Interaction Protocol 0-6 months (MBIP).^13^

*significance level *p*<0.05; **moderate to weak correlation; MS: maternal sensitivity; MI: maternal involvement; I: intrusiveness.

Table 4 presents the distribution of the score according to the categories of MBIP. It is noteworthy that the higher the score, the better the interaction between the mother-child dyad. There was no statistically significant difference between duration and frequency of Kangaroo Position and maternal sensitivity, maternal involvement, and intrusiveness. There was no statistically significant association between these maternal factors and the responses of the newborns in this sample (*p>*0.05).

**Table 4:**
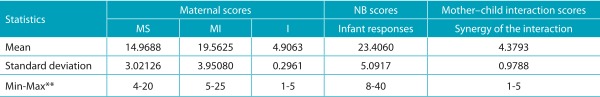
Distribution of the score according to the categories proposed by the Mother-Baby Interaction Protocol 0-6 months (MBIP).^15^

**According to the possible score of the items evaluated in the MBIP; MS: maternal sensitivity; MI: maternal involvement; I: intrusiveness; NB: newborn.

## DISCUSSION

The results showed that the Kangaroo Position favored the attempts of physical contact of the child with the mother during breastfeeding (*r*=0.37; *p*=0.03).

In relation to physical contact, the newborn needs to maintain the regulatory capacity to develop the interaction with the mother. When alert, the newborn makes attempts at verbal and physical contacts, which causes the mother to respond. This exchange of shifts in the future becomes a symbolic game and favors the acquisition and development of language, with the mother in a privileged position. In a study^15^ that aimed to analyze the waking state of infants aged one and five months, the authors observed that infants aged one month varied widely in the distribution of waking states. That is, this was the period of greatest variation of each specific regulatory state. This suggests that, at this stage of life, maintaining a waking state for a short period of time may influence the lesser availability to interact. These authors, when comparing term and preterm infants at six and nine months, observed that premature infants presented distancing (such as arching, pulling, twisting) from their mothers during filming, demonstrating that preterm infants have different regulation and interaction abilities than children born at term.

The newborns in this made more attempts at physical contact with the mother. The results of this research corroborate the study by Seidl-de-Moura et al., ^15^ in which the authors concluded that language also depends on the regulation system, since when there is more alert time, there is more possibility of establishing physical contact (gestural means), and communication (vocal and verbal means) with the mother. Therefore, newborns who stayed in the Kangaroo Position and made more attempts at physical contact, also had more opportunity to communicate with the mother, which may favor, along with other factors, the acquisition of language. In the study by Kritzinger and Rooyen,^17^ which aimed to determine the efficiency of communication training and its influence on the preterm infant-mother interaction, three groups were formed for evaluation:

1. with language training and Kangaroo Position practitioners.
2. without language training and Kangaroo Position practitioners.
3. no training and sporadic Kangaroo Position.

Groups 1 and 2, who practiced the Kangaroo Position, were those that had a positive effect on communication and mother-child interaction, demonstrated through a questionnaire, interview script on the knowledge of the children’s communication skills and behavioral video analysis of the infant-mother dyad interaction.

The results of this study were not significant in relation to the maternal sensitivity and the frequency of the Kangaroo Position, although all the newborns included evolved to breastfeeding. These findings contradict the results by Frota et al.,^18^ who observed that breastfeeding favored maternal sensitivity. However, when investigating the relationship between the type of infant feeding and mother-child interaction behaviors, based on the concept of maternal sensitivity, in three groups (preterm infants who needed intensive neonatal treatment, term infants who needed neonatal intensive care, and dyads with infants born at full term), Alfaya and Schermann^19^ inferred that maternal sensitivity is more favorable in mothers of full-term healthy infants (group with the highest rates of breastfeeding) than in mothers of infants born with neonatal risk.

In relation to immediate postpartum, different from what is recommended for healthy term newborns, no mother breastfed the child in the delivery room, and only one (3.4%) reported short skin-to-skin contact with the child. This may have happened due to prematurity, low weight or the need for some immediate ventilatory support. Thus, De Schuymer et al.,^20^ when comparing the development of the interaction abilities of term and preterm infants in the first year of life, observed that preterm infants, at six months of age, presented less provocation during face-to-face interaction and, at nine months, had fewer episodes of eye tracking. Therefore, it can be concluded that, in addition to immaturity, the perinatal experiences are different between term and preterm newborns, which may justify the possible delays in acquisitions and neuropsychomotor development in the latter.

When correlating the Kangaroo Position and the verbalization that the mothers performed during breastfeeding, data from this study revealed that the longer the Kangaroo Position, the less the mothers talked as the children (*r*=0.47; *p*=0.006).

The literature shows that the voice of the mother is part of an interactive cycle that can play an important role in the cognitive and social development of the child. A systematic review^21^ listed four main functions of maternal speech: affective communication, facilitation of social interaction, maintenance of child’s attention, and language acquisition. In another study,^15^ however, the mother’s speech with the 1-month-old son appeared to have a disruptive effect in 67% of the transitions (awake to restless), as it did not help the newborn to return to the awake (passive alert). On the contrary, it kept them in the restless state. It is believed that, in this study, the mothers verbalized less during breastfeeding because they perceived this disturbing effect, mainly associated to the high noise level of the neonatal units.^22^ In addition, it is worth mentioning the possibility that the camera inhibited the mothers at that time.

Excluding the dyads that did not perform the Kangaroo Position, the minimum frequency of this positioning was once and the maximum was 21 times. Regarding the duration of the Kangaroo Position, the minimum time in this group was 60 minutes, and the maximum time was 4,290 minutes. Therefore, the longer the hospital stay, the longer the mothers performed this position (*r*=0.36; *p*=0.01). This correlation was positive and statistically significant, probably due to the encouragement of some professionals or even the maternal presence in the neonatal unit. Regarding this, the literature points out that professionals working in Neonatology with early intervention are responsible for supporting the interpretation of children’s signals and teaching mothers how to help their children organize the regulatory activity.^23^ The adherence of mothers to the Kangaroo Method as a care technology for the family’s approximation and adaptation to the hospital context depends not only on the mother’s own will, but also on the support given by the family, the community, and the health team.^24^ There is a need to broaden the discussion on this topic with emphasis on women as participants in the process.^7^ ^,^ ^26^

A randomized clinical trial^27^ with preterm infants at six months of age demonstrated that intervening in mother-infant interaction is a promising approach to help preterm infants achieve the essential patterns of social interaction for optimal development. The maternal investment is a lucrative bet for development in the medium and long term.^28^ ^,^ ^29^ ^,^ ^30^

This study is the first, within the limits of our knowledge, to perform an early analysis of the interaction of the preterm infant-mother dyad and correlate it with the duration of the Kangaroo Position. This justifies the conflicting results between this study and other studies performed with infants at term or without risk for development or, even, in preterm infants after hospital discharge. However, there were some limitations, such as sample size and loss, the difficulty of the team adhering to not discarding Kangaroo Records during the cleaning of incubators, the noisy neonatal unit (a factor that interfered with camera audio, analysis of the footage and, possibly, the mother-child interaction), in addition to the presence of the camcorder. In spite of these limitations, it can be concluded with the present analysis of the mother’s interaction in the short term, after birth, that the greater time spent on the Kangaroo Position favored the attempts of physical contact of the child with the mother. This suggests a greater availability of the newborn to intensify the relationship with the mother during breastfeeding. The preterm newborn has the ability to go beyond feeding and have the time of breastfeeding as an opportunity to perform the initial contact exchanges.

Further research is needed to define the other patterns of preterm newborn interaction in order to allow the development of early intervention strategies. To this end, it is important that professionals involved in Neonatology focus their attention on mother-child relationships and be able to identify and intervene early in the difficulties of interaction during hospitalization. Investing in family-centered care is a promising strategy, in addition to using the Kangaroo Position as facilitator of exchanges between the mother-child dyad. However, it is necessary to move forward with regard to the quality of the Kangaroo Position in both infrastructure and ambience, as well as in terms of guidance and assistance to mothers, in order to encourage the occurrence of skin-to-skin contact with the child. It is worth mentioning that the ideal time for staying in the Kangaroo Position depends on the pleasure and comfort felt by the mother and the newborn, which demands adequate guidance and support from the local team.
